# Measurement of New Biomarkers of Immunity and Welfare in Colostrum and Milk of Pigs: Analytical Validation and Changes During Lactation

**DOI:** 10.3390/biology13100829

**Published:** 2024-10-16

**Authors:** María Botía, Damián Escribano, Eva Mainau, Alberto Muñoz-Prieto, José J. Cerón

**Affiliations:** 1 Interdisciplinary Laboratory of Clinical Analysis (Interlab-UMU), Veterinary School, Regional Campus of International Excellence ‘Campus Mare Nostrum’, University of Murcia, Campus de Espinardo s/n, Espinardo, 30100 Murcia, Spain; maria.botiag@um.es (M.B.); det20165@um.es (D.E.); jjceron@um.es (J.J.C.); 2 Department of Animal Production, Regional Campus of International Excellence ‘Campus Mare No Trum’, University of Murcia, Campus de Espinardo s/n, Espinardo, 30100 Murcia, Spain; 3 Department of Animal and Food Science, Autonomous University of Barcelona, Bellaterra, 08193 Barcelona, Spain; eva.mainau@uab.cat

**Keywords:** milk, colostrum, sow, pig, biomarkers, welfare, immune system

## Abstract

**Simple Summary:**

The colostrum and milk of sows may contain substances that could be used to evaluate immunity and welfare in pigs. This study aimed to quantify in these fluids four analytes associated with immunity: total adenosine deaminase (tADA) and its isoenzymes, myeloperoxidase (Mpx), calprotectin (S100A8/A9) and calgranulin (S100A12) and two analytes which are linked to welfare: alpha-amylase and cortisol. In addition, it aimed to evaluate how these analytes change during lactation and how they relate to each other and to immunoglobulin G (IgG) and immunoglobulin A (IgA). All the assays tested were able to quantify the analytes in a precise and accurate way. In addition, the analytes showed different dynamics through lactation, with adenosine deaminase being more concentrated in colostrum than in milk, while myeloperoxidase was more active in mature milk than in colostrum. Alpha-amylase was correlated with both IgG and IgA and S100A8/A9 was correlated with S100A12 and Mpx. Overall, the immune system and welfare analytes of this study can be measured in sow colostrum and milk samples and could have a potential use as biomarkers in these fluids.

**Abstract:**

Colostrum is a mammary secretion released from the time of farrowing to 36 h post-farrowing. After this time and during all the rest of lactation, the mammary secretion is considered milk. The objectives of this study were: (1) to perform an analytical validation in the colostrum and milk of sows of assays for four analytes related to immunity: total ADA (tADA) and its isoenzymes (ADA1 and ADA2), myeloperoxidase (Mpx), calprotectin, and calgranulin, and two analytes related to welfare: cortisol and alpha-amylase. (2) To evaluate the changes in these analytes during lactation (3) To assess the correlations between these new analytes, as well as with IgG and IgA. In the analytical validation, all the assays were precise and accurate. When changes during lactation were evaluated, the concentration of tADA and ADA2 was found to be higher in colostrum than in milk (*p* < 0.02), while the activity of Mpx was observed to be higher in mature milk than in colostrum (*p* < 0.03). Furthermore, cortisol and alpha-amylase activity were found to be higher in colostrum compared to mature milk (*p* < 0.04 and *p* < 0.0001, respectively). Regarding the relation between analytes, alpha-amylase showed a significant correlation with both IgG and IgA and calprotectin was correlated with calgranulin and Mpx. Further studies should be performed to elucidate the possible practical application of the analytes evaluated in this study as biomarkers of colostrum and milk in sows.

## 1. Introduction

Colostrum is a mammary secretion released from the time of farrowing to 36 h post-farrowing. After this time and during all the rest of lactation, the mammary secretion is considered milk [1]. Colostrum is characterized by its high concentrations of immunoglobulin G (IgG). Sow’s milk, in comparison with colostrum, has a reduced concentration of IgG and increased fat and lactose. For example, in a previous report, a mean concentration of IgG of 24.4 mg/mL was found in colostrum whereas concentrations of IgG in milk were 1.77 mg/mL [2,3]. IgG intake during the first few hours is essential for the growth and survival of piglets, as they are born agammaglobulinemic due to epitheliochorial placentation in pigs, while immunoglobulin A (IgA) is required throughout lactation to regulate the piglet intestinal microbiome, which is essential for proper digestive function [4,5].

When assessing sow’s colostrum and milk, IgG and IgA are the analytes more frequently measured. However, casein and 70 other proteins have been detected in colostrum using proteomic techniques [1], and various interleukins have been quantified [2]. This reflects that in colostrum and milk, there are components in addition to immunoglobulins that could be explored and analyzed to assess their potential as possible biomarkers to evaluate these fluids. Some of these components can be related to immunity and others with stress.

One of these immunity-related analytes is adenosine deaminase (ADA), an enzyme which is related to lymphocyte function and that can be measured in its total form (tADA), as well as two isoenzymes, adenosine deaminase 1 (ADA1) and adenosine deaminase 2 (ADA2), which can also be evaluated [6]. tADA has been measured in colostrum from other species different from the pig, such as cattle, suggesting that the activity of this enzyme may correlate with the number of T helper cells (CD4+) in colostrum and may be a potential indicator of immune status, providing information about cellular immunity [6]. Myeloperoxidase (Mpx) is also an enzyme related to the immune system, being mainly involved in neutrophil function, which has also been measured in milk from cows and showed increases in inflammation [7]. In addition, S100 proteins, such as calprotectin (S100A8/A9) and calgranulin (S100A12), are proteins associated with immune function, particularly the innate immune response, and can be quantified in colostrum and milk [8]. S100A12 has been studied in bovine milk and can be considered as a biomarker of mastitis [9]. Although S100A8/A9 has been measured in sow’s milk throughout lactation [10], to the author’s knowledge, ADA, Mpx, and S100A12 have not been measured in the colostrum or milk of sows.

Other biomarkers commonly associated with stress and welfare have previously been measured in colostrum and milk. A previous study in sows found no difference in cortisol concentrations, which are considered as a biomarker of hypothalamus–hypophysis activation, between colostrum and milk samples [11]. In humans, alpha-amylase activity, which, among other roles, is a biomarker of sympathetic activity, was found to be higher in colostrum than in milk, possibly to compensate for the lack of digestive enzymes at birth [12].

The hypotheses of this study are that there are new analytes related to immunity and welfare, in addition to IgG and IgA, that could be measured in sows’ colostrum and milk, and that they can show changes during lactation, with some of them potentially being related. Therefore, the objectives of this study were as follows: (1) to perform an analytical validation of assays for four analytes related to immunity: tADA and its isoenzymes (ADA1 and ADA2), Mpx, calprotectin and calgranulin, and two analytes related to welfare: cortisol and alpha-amylase, in the colostrum and milk of sows. (2) To evaluate the changes in these analytes during lactation. (3) To assess the correlations between these new analytes, as well as with IgG and IgA.

## 2. Materials and Methods

### 2.1. Animals, Housing and General Management

The samples used were from the control group of a previous report [2]. Therefore, a group of sixteen (Landrace × Duroc) multiparous sows randomly selected on the day of farrowing from a commercial farm (l’Heura S.L., Santa Perpetua de Mogoda, Barcelona, Spain) were used in this study. All sows employed in this study were subjected to clinical examinations prior to this study and continuously throughout the experiment, and no signs of disease were observed.

The sows were translated to the farrowing room on day 109 of gestation and were accommodated in farrowing crates (1.95 × 0.60 m), which were situated in the middle of farrowing pens (2.40 × 1.80 m). The floors were metal slatted. The animals had ten hours of light every day (7:00 to 17:00). The feeding of the sows was divided into twice a day, at 7:00 and 15:00, with an amount of 2.6 kg of feed per day, and they had ad libitum access to water.

Two injections of 1 mL, one at 7:00 and the other at 11:00, of Planate^®^ (Cloprostenol 0.092 mg/mL, MSD Animal Health, Friesoythe, Germany) were used on day 113 of gestation to induce the farrowing in the sows. Any manual interventions or treatments were performed following the normal routine of the farm and were always carried out by the same personnel. During farrowing, some of these routine treatments were the administration of 200 mg of vetrabutine hydrochloride (Monzal^®^, Boehringer Ingelheim España, S.A., Barcelona, Spain), if the cervical canal presented insufficient dilatation, or 1 mL of oxytocin (Hormonipra^®^, Hipra S.A, Gerona, Spain) if the interval time between two piglets was greater than 1 h. In addition, if the sow was very nervous around farrowing, the administration of Azaperone (Stressnil^®^, Janssen Animal Health, Elanco, Brussels, Belgium) or a beta blocker Carazolol (Suacron^®^, Divasa Farmavic S.A., Barcelona, Spain) was employed. All administrations were performed intramuscularly at the level of the neck.

### 2.2. Sampling Procedure

Colostrum samples were obtained at 24 h following farrowing (T1), while milk samples were obtained on the ninth and twentieth days following farrowing (T9 and T20, respectively) from all sows under study. Sows were injected with 0.7 mL IM of oxytocin (Hormonipra^®^, Hipra SA, Gerona, Spain) and, 30 s later, 2 mL of colostrum and milk samples were collected into sterile tubes. Then, colostrum and milk samples were directly frozen at −80 °C until analysis. Lactoserum was obtained from colostrum/milk after two 60 min centrifugations at 50,000× *g* at 4 °C.

In our experiments, there were no acquired animals because the procedures were performed in a commercial farm. The farm personnel oversaw all management activities under Universitat Autònoma de Barcelona (UAB) staff supervision. All procedures were also approved by the Institutional Animal Care and Use Committee of the UAB (CEEAH-1591) and the Generalitat de Catalunya (DMAH-6720).

### 2.3. Biochemical Analysis of Colostrum/Milk Samples

#### 2.3.1. Biomarkers of the Immune System

tADA was analyzed with a commercially available spectrophotometric automated assay (Adenosine Deaminase assay kit, Diazyme Laboratories, Poway, CA, USA) in an Olympus AU400^®^ Chemistry Analyzer (Olympus Diagnostics GmbH, Hamburg, Germany), which has been previously validated for pig saliva and serum [13].

In order to determine the specific isoenzyme, the inhibitor Erythro-9-(2-hydroxy-3-nonyl) adenine (EHNA) (Merck KGaA, Darmstadt, Germany) was employed. At an appropriate concentration, EHNA inhibits the ADA1 isoenzyme, while ADA2 remains unaffected [14]. Consequently, the tADA and ADA2 isoenzymes can be identified when samples are analyzed in the absence and presence of EHNA, respectively. The isoenzyme ADA1 was calculated as the difference between the two measurements, as previously described [13].

Mpx activity was measured by an automated assay, based on a previously described spectrophotometric manual assay [15], in an Olympus AU400^®^ Chemistry Analyzer (Olympus Diagnostics GmbH, Hamburg, Germany).

S100A8/A9 was analyzed with the BÜHLMANN fCal Turbo^®^ assay kit (BÜHLMANN, Laboratories AG, Schönenbuch, Switzerland) using an Olympus AU400^®^ Chemistry Analyzer. This kit has been previously validated for pig saliva and serum [16].

S100A12 was measured by a two-step direct sandwich assay developed using alphaLISA technology in 96-well plates with a total sample volume of 5 μL per well, previously optimized for pig saliva [17].

#### 2.3.2. Biomarkers of Stress

Salivary alpha-amylase activity was measured by a commercial method (α-Amylase, OSR6182, Beckman Coulter) in an Olympus AU400^®^ Chemistry Analyzer that has been validated for saliva [18].

Cortisol was measured by a commercial solid-phase, competitive chemiluminescence enzyme immunoassay (Cortisol, REF LKC01, Siemens Health Diagnostics, Deerfield, IL, USA) that has been validated for pig saliva [19].

### 2.4. Analytical Validation of the Methods for the Measurement of the Analytes of This Study

All methods used in this study (analysis of tADA, ADA2, Mpx, S100A8/A9, S100A12, alpha-amylase, and cortisol) were validated for colostrum from sows. The following validation characteristics were determined.

Imprecision. This was evaluated by the intra- and inter-assay coefficients of variation (CVs). Two samples containing high and low activities of any analyte were used. The intra-assay CV was determined by analyzing the samples five times in a single analytical run. For inter-assay precision, 5 aliquots from each pool were stored at −80 °C, and each was measured in duplicate on five different days, each time using freshly prepared standard curves. The CV was calculated as the percentage of the standard deviation (SD) of the replicates divided by the mean.

Accuracy. This was indirectly investigated by linearity under dilution. This was determined using a serially diluted sample (1:2, 1:4, 1:8, 1:16 and 1:32) with the assay buffer. Afterwards, linear regression between the observed and the expected results was performed and the R^2^ was calculated.

Sensitivity. The limit of detection (LD) was determined to assess the sensitivity of the method. The LD was calculated as the mean of 15 replicate measurements of the assay buffer plus three SDs.

### 2.5. Changes in the Analytes in Lactation

All the analytes were measured in the colostrum samples obtained on the first day following farrowing (T1) and in all milk samples were obtained on the ninth and twentieth days following farrowing (T9 and T20, respectively).

### 2.6. Statistical Study

The analysis of the method validation (means, SDs, CVs, and linear regression) was performed using routine descriptive statistics procedures (Excel 2019, Microsoft 11). Statistical analysis was performed and graphs of the results obtained were created using Graph Pad software (GraphPad Prism, version 9 for Windows, Graph Pad Software Inc., San Die-go, CA, USA).

The normality of the data was assessed using the Shapiro–Wilk test, showing a non-normal distribution. To determine any significant differences in each analyte concentration obtained each time, Friedman’s test, followed by Dunn’s multiple comparison test, was applied. Significance was set at *p* < 0.05. The correlations between each analyte evaluated and with IgG and IgA were assessed using Spearman’s correlation coefficients. IgG and IgA data were obtained from a previous report [2].

## 3. Results

### 3.1. Analytical Validation

All methods provided intra- and inter-assay CVs lower than 15% (Table 1 and Table 2). Linear regression analysis provided an R^2^ higher than 0.98 in all assays when linearity after serial sample dilution was assessed (Table 3 and Table 4). The LD for tADA and ADA2, S100A12, alpha-amylase, and cortisol were set at 0.07 IU/L, 0.000002 mg/L, 11.65 IU/L and 0.05 μg/dL, respectively. The LD of Mpx and S100A8/A9 could not be calculated since all measurements with ultrapure water gave a value of zero.

### 3.2. Changes in Lactation

**Figure 1:**
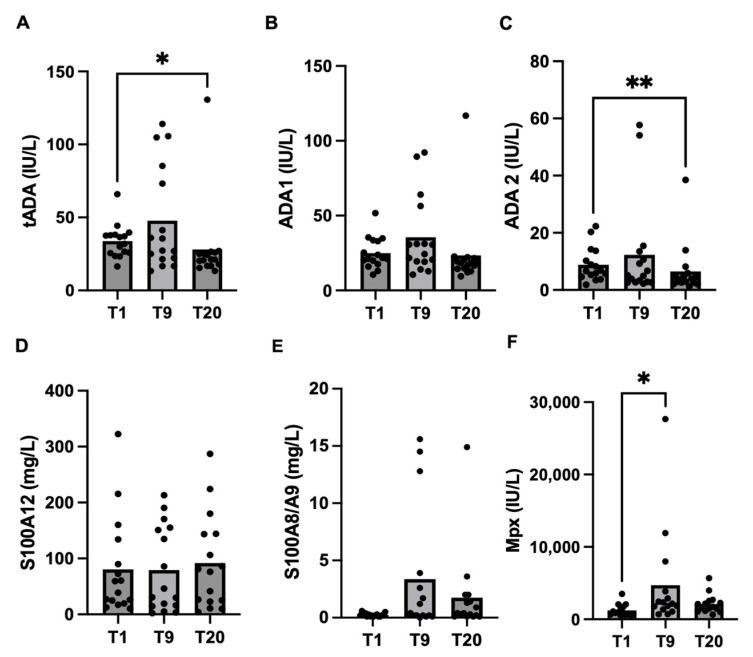
Changes in tADA (**A**), ADA1 (**B**), ADA2 (**C**), S100A12 (**D**), S100A8/A9 (**E**) and Mpx (**F**) in colostrum/milk samples obtained at one day (T1), nine days (T9) and twenty days (T20) after farrowing. Asterisks indicate significant differences (** *p* ≤ 0.01; * *p* ≤ 0.05).

In the immune-related biomarkers, tADA, ADA2 and Mpx showed significant differences between colostrum and milk. tADA concentrations were significantly (*p* = 0.02) lower in milk (T20) (median: 21.3 IU/L; 25–75th percentile: 17.1–25.8 IU/L) compared to colostrum (T1) (median: 34.3 IU/L; 25–75th percentile: 25.7–38.1 IU/L). ADA2 also showed significantly higher values at T1 (median: 6.7 IU/L; 25–75th percentile: 4.7–12.8 IU/L) compared to T20 (median: 3.8 IU/L; 25–75th percentile: 2.6–5.5 IU/L) (*p* = 0.005) Mpx showed significantly higher values at T9 (median: 2171 IU/L; 25–75th percentile: 1357–3853 IU/L) compared to T1 (median: 901 IU/L; 25–75th percentile: 697–1739 IU/L) (*p* = 0.03). S100A12 and S100A8/A9 showed no significant changes between the days of sampling. These results are shown in Figure 1 and Table 5.

**Figure 2:**
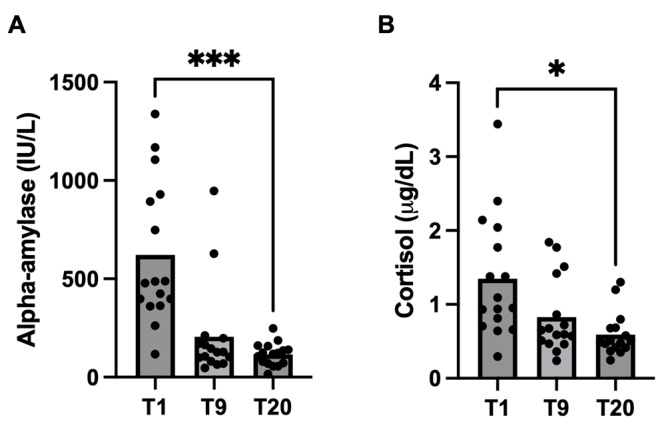
Changes in alpha-amylase (**A**) and cortisol (**B**) concentrations in colostrum/milk samples obtained at one day (T1), nine days (T9) and twenty days (T20) after farrowing. Asterisks indicate significant differences (*** *p* ≤ 0.001; * *p* ≤ 0.05).

Stress biomarkers showed significantly higher values at T1 than at T20 (*p* < 0.05). Cortisol concentrations in colostrum (T1) (median: 1.02 μg/dL; 25–75th percentile: 0.7–1.97 μg/dL) were significantly (*p* = 0.04) higher than in milk (T20) (median: 0.49 μg/dL; 25–75th percentile: 0.41–0.68 μg/dL). Alpha-amylase also showed significantly (*p* = 0.0001) higher values at T1 (median: 482 IU/L; 25–75th percentile: 372–920 IU/L) than at T20 (median: 120 IU/L; 25–75th percentile: 69.9–153 IU/L). These results are shown in Figure 2 and Table 6.

### 3.3. Correlation between Analytes and IgG and IgA

Alpha-amylase showed a significant positive correlation with IgG (r = 0.68; *p* value < 0.0001), IgA (r = 0.5; *p* value < 0.0001) and tADA (r = 0.60; *p* value < 0.0001). tADA was also positively correlated with its two isoenzymes ADA1 (r = 0.88, *p* value < 0.0001) and ADA2 (r = 0.70, *p* value < 0.0001). In addition, S100A8/A9 correlated significantly and positively with Mpx (r = 0.63; *p* value < 0.0001) and S100A12 (r = 0.69; *p* value < 0.0001).

## 4. Discussion

This study has demonstrated that there are four biomarkers of immunity (tADA and its isoenzymes, Mpx, S100A12, and calprotectin) and two analytes related to stress (cortisol and alpha-amylase) that can be measured in the colostrum and milk of sows and that show different dynamics during lactation. To the author’s knowledge, this is the first report in which Mpx, tADA and its isoenzymes, S100A12 and alpha-amylase have been measured in sows’ colostrum/milk samples and their possible relations with calprotectin and cortisol studied.

The assays used for the measurement of all the analytes of this study were found to be highly precise, as all showed an inter- and intra-assay CV of less than the 20% recommended [20]. In addition, the R^2^ of linearity under serial sample dilution was close to 1 for all methods, indicating their accuracy [21]. It is interesting to point out that, with the exception of S100A12, all analytes were measured by automated assays, which provides advantages from the technical point of view, such as higher precision and sample throughput. However, the assays of this study can also be adapted to other assay formats, such as an ELISA or manual spectrophotometers; therefore, they can easily be set up in the laboratory.

The validated assays were applied to study and evaluate the possible changes in the biomarkers of this study in colostrum samples collected one day (T1) after farrowing and in milk samples obtained nine days (T9) and twenty days (T20) after farrowing. In order to obtain a representative sample, colostrum samples were taken 24 h after farrowing. This timeframe was selected to avoid the potential for the sample to be close to the limit between colostrum and milk, which is described to occur 36 h after farrowing. Regarding immune-related biomarkers, the results demonstrated significantly higher tADA concentrations in colostrum (T1) compared to mature milk (T20). These findings are in accordance with those previously reported in other species, with tADA concentrations in bovine colostrum being higher than in milk [6]. Given that tADA is an enzyme predominantly expressed in lymphoid tissue, the elevated number of lymphoid cells observed in colostrum compared to mature milk may provide a potential explanation for the high values of tADA found in the colostrum in our study [22,23]. tADA has been described to be related to lymphocyte T function, and in cows’ colostrum, a correlation between tADA and the number of CD4+ T cells in the colostrum was found [5]. This could indicate that tADA might have the potential to assess cell-mediated immunity in colostrum, being a potentially appropriate complement to the measurement of IgG, which only assesses humoral immunity.

Mpx showed a significant increase in milk (T9) compared to the colostrum (T1) sample. Mpx is an enzyme that is present in high concentrations in neutrophils. It is released in situations of inflammation and requires the presence of H₂O₂ in order to engage in its biological activity [24,25]. In a study conducted on humans revealed that the polymorphonuclear cells present in colostrum are hypofunctional. This reduces oxygen metabolism and, consequently, the amount of available H₂O₂ required for other enzyme systems, such as Mpx, to perform their functions [26]. This finding may be the reason for the reduced levels of Mpx observed in colostrum relative to mature milk in the present study. A previous study observed an increase in Mpx in cows with mastitis compared to healthy cows, indicating its potential as a marker of inflammation [7]. Considering these findings, further research is recommended to ascertain the function of Mpx in porcine milk and its potential application as an inflammatory biomarker.

The S100 family is mainly associated with innate immune responses. S100A12 and S100A8/A9 showed no significant differences in milk between the different time points of lactation in our study. A previous report conducted on colostrum and milk from swine observed low levels of S100A8/A9 in colostrum, with a subsequent increase in concentration over the course of the following days [10]. Although the results of the present study are not statistically significant, a similar trend in S100A8/A9 concentrations was observed, with an increase being seen at nine days post-farrowing. This increase may be related to the inflammation of the mammary gland caused after the first days of suckling by the piglets. In other species, such as cattle, the S100A12 protein has been detected by proteomic analysis in the milk of *E. coli*-infected cows, whereas this protein was not found in healthy cows [27]. Interestingly, S100A8/A9 and S100A12 proteins were found to be correlated, which could be explained by the fact that both belong to the same protein family. Additionally, S100A8/A9 demonstrated a positive correlation with Mpx, which can be attributed to the fact that both analytes are released by neutrophils in situations of inflammation [28]. Further studies are recommended to evaluate the potential of the two S100 proteins analyzed in this study as biomarkers of innate immunity and inflammation in colostrum and milk.

Biomarkers related to stress also showed differences between colostrum and milk. In the case of cortisol, significantly higher concentrations were observed in colostrum (T1) than in mature milk (T20). This differs from the results of a previous study, where no significant changes in cortisol levels were observed between colostrum and milk samples [11]. However, other studies have observed increases in salivary and hair cortisol in sows around and after parturition [29,30]. Given the lipophilic nature of glucocorticoids, cortisol can pass into milk by simple diffusion [31]. Therefore, the increase in cortisol in the systemic circulation after farrowing [32] could be the reason for the increase in cortisol in colostrum. From the point of welfare evaluation, it would be of interest to evaluate two aspects regarding cortisol in sow colostrum and milk. One factor to consider is the influence of previous stress of the sow on cortisol concentrations in milk. For example, in humans, previous stressful experiences in mothers could lead to variations in breast milk cortisol and affect the amount of cortisol transferred to infants through breast milk [33]. The second consideration is the effect of varying cortisol concentrations in milk on piglets’ behavior. This topic has been studied in animal models with controversial results since, for example, in monkeys, higher concentrations of cortisol in milk have been associated with more frequent play and social behavior [34], whereas in another study, higher breast milk cortisol concentrations were related to a more nervous and less confident temperament [35].

The alpha-amylase concentration was found to be significantly higher in colostrum (T1) than in mature milk (T20), a finding consistent with previous reports on human colostrum and milk [36] as well as in cows [37]. The elevated alpha-amylase levels observed in colostrum relative to milk may be attributed to a compensatory mechanism to offset the reduced pancreatic enzyme activity in the piglet, given the necessity for postnatal pancreatic maturation [38]. A high variability was found in alpha-amylase activity values in the sows’ colostrum in our study; this high inter-individual variation has also been described in human colostrum [12].

It is interesting to point out that, in the present study, there are correlations between the different analytes and IgG and IgA concentrations. Alpha-amylase showed a moderate positive correlation with both IgG and IgA. This correlation may be attributed to the tendency of alpha-amylase to bind to globulins [39]. Indeed, in cases of systematic diseases, macroamylasaemia is prevalent when IgG is elevated [40]. Further studies should be conducted to elucidate if alpha-amylase could be a complement to the measurement of IgG in sow colostrum.

This study has several limitations. Ideally, the validation of the assays should have also included an evaluation of accuracy by comparison with other validated methods or by recovery tests of the purified proteins. However, the lack of available assays and purified material of these proteins did not allow these evaluations. In addition, the changes in lactation were evaluated in a limited number of animals. In the future, it would be desirable to perform further studies with a larger number of individuals to confirm these findings. Also, further studies should be performed to evaluate the potential of the different analytes as biomarkers of aspects that are usually not evaluated in colostrum, for example, the potential of ADA as a marker of cell immunity, Mpx as a biomarker of neutrophil function, S100s as biomarkers of the innate response and cortisol as a biomarker of welfare.

## 5. Conclusions

Overall, it can be concluded that the biomarkers of the immune system, ADA, Mpx, S100A8/A9 and S100A12, and the biomarkers of welfare, alpha-amylase and cortisol, can be measured in sows’ colostrum and milk samples. These analytes have different dynamics during lactation, with tADA, ADA2, alpha-amylase and cortisol having higher values in colostrum, whereas Mpx had higher values in milk, and S100 A8/A9 and S100A12 did not show significant changes. These results open new possibilities for the potential application of biomarkers in sow’s colostrum and milk, and further studies should be performed to elucidate this.

## Figures and Tables

**Table 1:** Mean, standard deviation (SD), and coefficient of variation (CV) obtained in the imprecision study for assay validation of biomarkers related to the immune system in colostrum samples. tADA (total adenosine deaminase), ADA2 (adenosine deaminase 2), Mpx (myeloperoxidase), S100A8/A9 (calprotectin) and S100A12 (calgranulin).

Analyte	Mean	SD	CV (%)
**tADA (IU/L)**			
Intra-assay			
High concentration	224.1	4.9	2.2
Low concentration	29.4	0.6	2
Inter-assay			
High concentration	206.4	15.7	7.6
Low concentration	30.6	0.7	2.3
**ADA2 (IU/L)**			
Intra-assay			
High concentration	47	4.9	10.5
Low concentration	13.5	0.9	6.8
Inter-assay			
High concentration	46.2	5	10.9
Low concentration	13	1.4	10.7
**Mpx (IU/L)**			
Intra-assay			
High concentration	5076	16.5	1.4
Low concentration	669.3	13.5	2
Inter-assay			
High concentration	4629	754.2	16.2
Low concentration	606.4	82.4	13.6
**S100A8/A9 (mg/L)**			
Intra-assay			
High concentration	1.42	0.005	0.4
Low concentration	0.11	0.005	4.9
Inter-assay			
High concentration	1.44	0.05	3.2
Low concentration	0.18	0.01	7.8
**S100A12 (mg/L)**			
Intra-assay			
High concentration	1155	38.8	3.4
Low concentration	157.9	10.3	6.5
Inter-assay			
High concentration	1072	82.7	12.9
Low concentration	140.9	13.1	9.3

**Table 2:** Mean, standard deviation (SD) and coefficient of variation (CV) obtained in the imprecision study for assay validation of biomarkers related to welfare in colostrum samples.

Analyte	Mean	SD	CV (%)
**Alpha-amylase (IU/L)**			
Intra-assay			
High concentration	1196	16.5	1.4
Low concentration	401.5	1.9	0.5
Inter-assay			
High concentration	1181	11.3	1
Low concentration	401.7	6.4	1.6
**Cortisol (μg/dL)**			
Intra-assay			
High concentration	3.24	0.12	3.57
Low concentration	0.6	0.1	10.4
Inter-assay			
High concentration	3.51	0.35	9.9
Low concentration	0.58	0.06	10.4

**Table 3:** Linear regression analysis between expected and observed results from a linearity of dilution study of biomarkers related to the immune system in colostrum samples. tADA (total adenosine deaminase), ADA2 (adenosine deaminase 2), Mpx (myeloperoxidase), S100A8/A9 (calprotectin) and S100A12 (calgranulin).

Analyte	Value	Slope	Y-Intercept	R^2^
**tADA (IU/L)**				
High concentration	229.2	1	11.2	0.99
Low concentration	28.8	1.09	3.17	0.99
**ADA2 (IU/L)**				
High concentration	47.2	1	0.55	0.99
Low concentration	13.5	0.93	0.98	0.99
**Mpx (IU/L)**				
High concentration	4927	0.98	167.9	0.99
Low concentration	661.5	0.98	16.85	0.99
**S100A8/A9 (mg/L)**				
High concentration	1.42	0.97	0.06	0.95
Low concentration	0.23	0.84	0.02	0.99
**S100A12 (mg/L)**				
High concentration	1613	0.99	22.27	0.99
Low concentration	139.8	1	0.23	0.99

**Table 4:** Linear regression analysis between expected and observed results from linearity of dilution study of biomarkers related to welfare in colostrum samples.

Analyte	Value	Slope	Y-Intercept	R^2^
**Alpha-amylase (IU/L)**				
High concentration	1215	1	31.9	0.99
Low concentration	400.8	0.99	5.53	0.99
**Cortisol (μg/dL)**				
High concentration	3.24	0.97	0.16	0.99
Low concentration	0.72	0.97	0.03	0.99

**Table 5:** Median, standard deviation (SD), standard error of the mean (SE), and *p* value of biomarkers related to the immune system in colostrum/milk samples obtained at one day (T1), nine days (T9), and twenty days (T20) after farrowing.

Analyte/Time	Median	SD	SE	*p*-Value *
**tADA (IU/L)**				
**T1**	34.3	11.4	2.9	
**T9**	31.5	35.8	8.9	>0.99
**T20**	21.3	27.7	6.3	0.02 ^a^
**ADA1 (IU/L)**				
**T1**	21.5	10.4	2.6	
**T9**	27.4	25.9	6.5	>0.99
**T20**	18.35	25.2	6.3	0.23
**ADA2 (IU/L)**				
**T1**	6.7	5.9	1.5	
**T9**	5.0	17.5	4.4	0.75
**T20**	3.8	9.1	2.3	0.005 ^a^
**Mpx (IU/L)**				
**T1**	901	824	212	
**T9**	2171	7033	1816	0.03 ^b^
**T20**	1749	1816	333	0.05
**S100A8/A9 (mg/L)**				
**T1**	0.2	0.2	0.004	
**T9**	0.3	5.6	1.4	0.1
**T20**	0.4	3.6	0.9	0.2
**S100A12 (mg/L)**				
**T1**	49.1	87.5	21.9	
**T9**	38.2	76.4	19.1	>0.99
**T20**	78.6	84.2	21.1	>0.99

* *p* value represents the statistical significance of the comparison between T9 and T20 with respect to T1. ^a^ indicates a decrease compared to T1. ^b^ indicates an increase compared to T1. No significant differences were found between T9 and T20.

**Table 6:** Median, standard deviation (SD), standard error of mean (SE) and *p* value of biomarkers related to welfare in colostrum/milk samples obtained at one day (T1), nine days (T9) and twenty days (T20) after farrowing.

Analyte/Time	Median	SD	SE	*p* Value *
**Alpha-amylase (IU/L)**				
**T1**	482	360	90.0	
**T9**	128	239	59.8	0.06
**T20**	120	57.9	14.5	0.0001 ^a^
**Cortisol (μg/dL)**				
**T1**	1.0	0.8	0.2	
**T9**	0.6	0.5	0.1	>0.99
**T20**	0.5	0.3	0.1	0.04 ^a^

* *p* value represents the statistical significance of the comparison between T9 and T20 with respect to T1. ^a^ indicates a decrease compared to T1. No significant differences were found between T9 and T20.

## Data Availability

The original contributions presented in the study are included in the article, further inquiries can be directed to the corresponding author.
